# Digital Protocol to Record Occlusal Analysis in Prosthodontics: A Pilot Study

**DOI:** 10.3390/jcm13051370

**Published:** 2024-02-28

**Authors:** Emanuele Risciotti, Nino Squadrito, Daniele Montanari, Gaetano Iannello, Ugo Macca, Marco Tallarico, Gabriele Cervino, Luca Fiorillo

**Affiliations:** 1 Private Practice, 20145 Milan, Italy; risciotti.e@tiscali.it; 2 Private Practice, 16121 Genova, Italy; nino.squadrito@libero.it (N.S.); d.montanari19@gmail.com (D.M.); 3 Private Practice, 16036 Genova, Italy; gaetano.iannello@micerium.it; 4 Private Practice, 96100 Siracusa, Italy; ugomaccast@gmail.com; 5 School of Dentistry, University of Sassari, 07100 Sassari, Italy; me@studiomarcotallarico.it; 6 Department of Biomedical and Dental Sciences and Morphofunctional Imaging, University of Messina, 98122 Messina, Italy; gabrielecervino@gmail.com; 7 Department of Dental Cell Research, Dr. D. Y. Patil Dental College and Hospital, Dr. D. Y. Patil Vidyapeeth, Sant-Tukaram Nagar, Pimpri, Pune 411018, India; 8 Multidisciplinary Department of Medical-Surgical and Odontostomatological Specialties, University of Campania “Luigi Vanvitelli”, 80121 Naples, Italy

**Keywords:** intraoral scanning, occlusal analysis, CAD/CAM, Patient Specific Motion, prosthetic rehabilitation

## Abstract

**Background:** Digital technologies enable the accurate replication of occlusion, which is pivotal for stability in maximum intercuspation and dynamic occlusion. CAD softwares generates standardized occlusal morphologies requiring significant adjustments. The consideration of individual mandibular movements during restoration leads to better functional integration. This pilot study evaluates the efficacy of a novel, fully digital protocol for occlusal analysis recording in prosthodontics. **Methods:** Patients needing single or multiple metal-free restorations were included. Teeth underwent horizontal finish line preparation, while restorations on implants were either directly screwed or used multi-unit abutments. A digital impression (Trios 3 Intraoral Scanner) captured the mouth’s elements. Dynamic occlusion was recorded via Patient Specific Motion (PSM). After the placement and functionalization of temporary restorations, subsequent scans included various elements, and CAD software (Dental system) was used for the restoration design. Restorations were milled in monolithic zirconia, pressed from CAD/CAM-milled wax, and sintered. **Results**: An evaluation of 52 restorations in 37 patients indicated high accuracy in restorations manufactured via the fully digital workflow. Monolithic zirconia was predominantly used. Subtractive (17.3%) and additive (7.7%) occlusal adjustments were mainly chairside. **Conclusion:** This study underscores the efficacy of meticulous verification measures and a centric contact system in reducing the need for clinical occlusal refinements in prosthetic restorations.

## 1. Introduction

Intraoral scanning (IOS) systems have achieved significant reliability in accuracy and precision and have had widespread use in dental practice in recent decades [1]. CAD/CAM technology made fabricating dental and implant-supported restorations possible through a digital workflow. Digital impressions transfer the intraoral situation to a virtual model and represent the first step of the digital workflow. The accuracy of this procedure is crucial to transferring the implant position correctly, and it represents the success of the treatment. If it is performed poorly, it can lead to mechanical and biological complications. Digital impressions can accelerate the data-capturing process and eliminate most drawbacks usually found with conventional impressions, thereby decreasing patient discomfort while improving the predictability of prosthesis design and manufacturing procedures [2].

A recent systematic review demonstrated that the precision and accuracy of the digital workflow, compared with the conventional technique, favored up to four-unit restorations [3].

Precision is defined as the ability to take the same measurement value consistently [4]. An intraoral scanner should present high trueness and precision, and it can be evaluated by superimposing different scans of the same object using the same IOS device [5]. Many factors might compromise the performance of an IOS and decrease its accuracy. The aspects related to the equipment, such as the scanning technology, the state of the device, and the temperature and illumination of the room and the reading area, may affect the accuracy of the readings. Also, the operator’s skills, experience, and scanning technique are accuracy-influencing factors. In vivo, the patient’s movements, limited mouth opening, and oversized tongues may make the scanning procedure difficult. In vitro, the design and material of the cast and the design of the scan body, as well as its light reflection properties, can affect the precision of the digital impression [6,7,8].

Among the benefits of digital technologies is that occlusion can be accurately replicated using an IOS.

Occlusal design plays a significant role in maintaining and promoting stability in maximum intercuspation without generating interference in dynamic occlusion [9]. The digital workflow allows one to send information about the three-dimensional shape of the prepared tooth and adjacent and antagonist teeth, allowing for further CAD/CAM (computer-aided design/computer-aided manufacturing) processing of the prosthetic restoration [10]. However, CAD software generates occlusal morphologies based on standardized shapes requiring major occlusal adjustments [11,12]. For this purpose, using an articulator to simulate the movements of a working model is considered an indispensable aspect for prosthetic restorations [13]. Esposito et al. [14] investigated the reliability of recording occlusal contacts using an intraoral scanner versus articulating paper, finding significant differences in contact numbers except for upper central incisors and first premolars, with low clinician agreement on occlusions, highlighting the need for a precise method for recording occlusal contacts. Abbas et al. [15] studied the influence of occlusal reduction design on the biomechanics of endocrowns in maxillary premolars, revealing that PEKKTON endocrowns with anatomical preparations offer optimal restoration, suggesting these innovative systems could improve the longevity of tooth restorations. Pereira et al. [16] assessed the accuracy and reproducibility of real versus virtual occlusal contact points in implant-supported dentures, finding that both methods provided clinically excellent contact points with no significant difference in reproducibility, indicating intraoral scanners as a viable tool for occlusion mapping.

**Figure 1:**
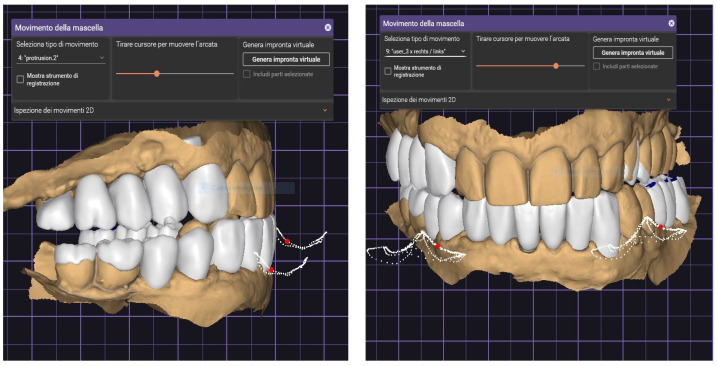
Protrusive movement (**left**) and gothic arch (**right**). These are the free movements of the patient. In this phase, verifying the accurate functional movements with the articulation paper previously detected on the patient is possible.

It has been demonstrated that the functions performed by a virtual articulator are comparable to those performed by an analog system [17]. However, to develop movements compatible with mandibular kinematics, analog models or digital scans must be positioned appropriately [18]. Analogically, this step is performed using an arbitrary or kinematic facebow, setting the condylar parameters, respectively, to mean values or according to pantographic tracings [18]. In a digital environment, models can be aligned using articulatory scanning with arch-mounted models [19] or by aligning STL models based on CBCT [20] or face scans [21], or by using jaw motion detection systems such as Arcus Digma or Zebris (Figure 1) [22], recording the individual parameters to be transferred to the virtual articulator. Digital technologies have recently been introduced, allowing mandibular movements to be acquired and reproduced in a virtual environment without needing to place them in a virtual articulator. 

Restorations fabricated with knowledge of individual mandibular movements have been reported to have better functional integration than restorations fabricated using medium articulator settings (Figure 1) [23]. 

For this purpose, the 3Shape system, combined with the trio’s scanner, allows for mandibular movements to be acquired through a function named Patient Specific Motion (PSM), with the possibility of reproducing it in the CAD environment to allow for the design of ideal prosthetic restorations according to actual mandibular movements and function. This pilot study aims to demonstrate and evaluate the efficacy of this digital procedure in recording an occlusal analysis.

## 2. Materials and Methods

The present pilot study was designed as a clinal audit to evaluate a novel, fully digital protocol for recording occlusal analyses through a case series. This study was conducted between January 2023 and May 2023. Patients who needed a single or up to a three-unit metal-free (zirconia or lithium disilicate) restoration delivered on natural teeth or implants were considered eligible for this study. Patients requiring complex occlusal therapy (re-organizational approach in centric relation and/or variation in the vertical dimension of occlusion) were excluded. Natural teeth were prepared with a horizontal finish line. At the same time, all the restorations on implants were screwed directly on the implants (single crown) or using a multi-unit abutment (MUA) if splinted. All the restorations were made starting with an IO scan of the patient’s mouth (Trios 3 Intraoral Scanner, 3Shape A/S, Copenhagen, Denmark). Then, the patient’s mandibular movements (dynamic occlusion) were recorded using the Patient Specific Motion (PSM) tool (3Shape A/S). All patients were rehabilitated in maximal intercuspidation. According to the Council for International Organization of Medical Sciences (CIOMS-2016), approval by an ethical committee was not required because “the research poses no more than minimal risk to participants” with this type of non-invasive intraoral scanning. The patients were selected among patients already candidates for prosthetic rehabilitation, no personal data are shown, and this method could not have caused any damage; in the case of an incompatible prosthesis, the patient would have continued with their temporary prosthetic rehabilitation before receiving a new prosthetic product.

### 2.1. Clinical Steps

Eligible patients underwent an initial scanning of the elements to be rehabilitated. In a second appointment, temporary restorations were applied and made functional on both teeth and implants. After four to six weeks of function, all the patients received the following scans: working arch with functionalized temporary restoration, definitive abutment or scan body, antagonist, right and left occlusions (scan bite), and PSM. Before carrying out the scans, the excursive occlusal contacts (protrusion and laterality) were marked using a 21 µ red articulating paper (Accufilm II red). In contrast, contacts in maximum intercuspidation were marked with 21 µ black articulating paper (Accufilm II black) so that clinical evidence of these areas was acquired during the color scanning phase to allow for contact verification during the CAD phases of making the restorations. After the first scan, the interim restoration was removed, and the second scan was taken at the implant level (scan body) or natural tooth (double-cord technique). Subsequently, antagonist and occlusion were recorded. Finally, a dynamic occlusion was scanned and recorded during the digital impression procedure in the PSM scan step. After that, all scans were sent to the laboratory via the “Communicate” intra-net system in the proprietary 3ox format (3Shape A/S).

### 2.2. Laboratory Steps

**Figure 2:**
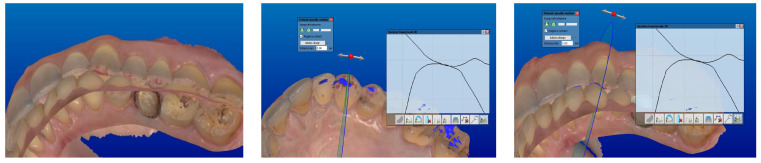
Checking the occlusion and the mandibular movements.

**Figure 3:**
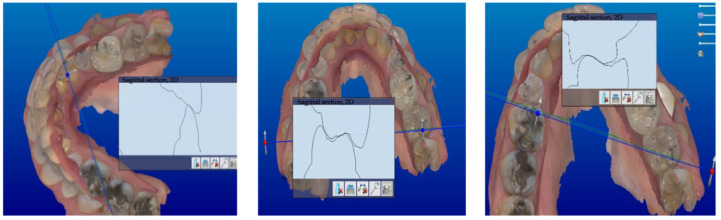
Sagittal view corresponding to the marked area.

**Figure 4:**
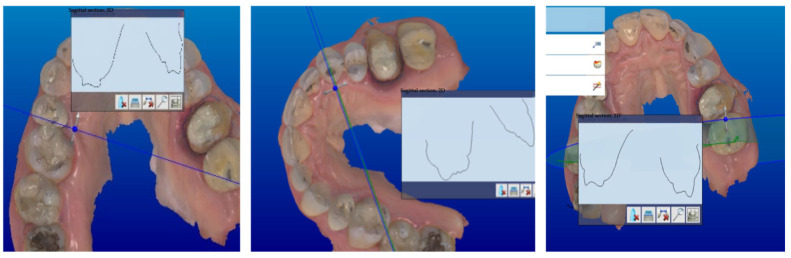
Marked contacts.

All the scans were imported into CAD software (Dental system). The accuracy of the intermaxillary relationships was verified at the sagittal view, corresponding to the areas clinically marked with the articulation paper, to verify the absence of encroachments or spacings, both in maximum intercuspidation and in excursive movements (Figure 2, Figure 3 and Figure 4).

**Figure 5:**
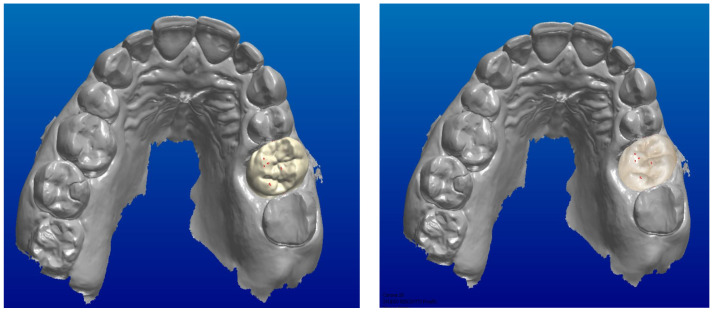
Reinforced occlusal contact points using an additive knife tool.

After that, the aesthetic–functional project of definitive restorations was carried out by reproducing an ideal anatomical wax-up according to the Geometric Functional Anatomy (AFG) technique, replacing the use of a caliper with a 3D grid that provided anatomical references. After a careful verification of the occlusal morphology and functional movements, the occlusal contacts in MI were reinforced with the individual morphing tool, using a radius with a 0.48 mm diameter and a level of influence with a thickness of 25 μ using the “additive wax knife tool” (Figure 5).

Definitive restorations were milled in monolithic zirconia 850, using cutting tools with a 0.2 mm diameter, and subsequently sintered according to the manufacturer’s recommendations. The lithium disilicate restorations were pressed starting from CAD/CAM-milled wax and finally sintered according to the manufacturer’s recommendations (Table 1).

Finally, all the restorations were finished and polished, maintaining the reinforced points under protection. After sintering, the interproximal and occlusal contacts were marked with a pencil to avoid contact with the bur and polishing rubbers. All the phases were performed fully digitally, without the need to create any master models. The CAD parameters are reported in Table 2.

Once in the dental office, an intraoral check of the interproximal contacts and the internal fit of the restorations was performed using a fit checker. After that, the occlusal contacts were checked in the same way as previously described, using 21 µ red articulating paper (Accufilm II red), while the contacts in maximum intercuspidation were marked with 21 µ black articulating paper (Accufilm II black). Moreover, 8 µ Shimstock (company) paper was used to check all the contacts. 

Occlusal verification was carried out before cementation or for the implants after the verification of passivity and tightening the screws. The present research recorded and analyzed the number and type of occlusal adjustments. Periapical radiographs were obtained if needed.

## 3. Results

A total of 52 new restorations and not remakes, delivered on 37 patients, were evaluated. All the restorations were made in MI using lithium disilicate or monolithic zirconia. All the restorations were made starting from an intraoral digital impression and patient-specific motion acquisition, according to a fully digital workflow.

On thirty-three patients, definitive restorations were made in monolithic zirconia, while lithium disilicate was used in the other four. A total of forty single crowns were delivered; of these, eight were delivered on implants and bonded on T-base abutments. A total of 12 restorations were multiple. Of these, three bridges of three units each were delivered on natural teeth, and only one was delivered on implants (Table 2). Restorations were applied on incisors and premolar and molar teeth. All multi-unit rehabilitations were performed on premolar–molar teeth.

A total of nine subtractive occlusal finishings (17.3%) and four addictive occlusal finishings (7.7%) were performed. All the subtractive occlusal adjustments were made chairside, while all the four addictive occlusal finishings were made in the laboratory. In this case, the crowns were delivered in later appointments (Table 3).

- Study participants: 52 restorations were evaluated across 37 patients.
- Materials used: restorations primarily used monolithic zirconia.
- Occlusal adjustments: 17.3% of the cases required subtractive occlusal finishing, and 7.7% required additive finishing.
- Finishing techniques: subtractive occlusal adjustments were performed chairside, whereas all additive occlusal finishings were executed in the laboratory.

These results highlight the efficacy and precision of the fully digital workflow in prosthetic dental restorations, emphasizing the reduced need for post-production adjustments.

## 4. Discussion

The present study was designed as a clinical audit to evaluate the efficacy of a novel, fully digital protocol for recording occlusal analyses. The preliminary results encourage the presented protocol, improving the final accuracy of the restorations and reducing the need for finishing. This study compares the new digital method with traditional methods. An occlusal analysis in prosthodontics traditionally involves physical impressions and manual adjustments to replicate patient-specific occlusal dynamics. This process can be time-consuming and less precise, often requiring several adjustments to achieve ideal occlusion. Using wax for an occlusal analysis in prosthodontics has several disadvantages. Wax impressions can be less accurate due to distortion or deformation during handling or storage. The process is also time-consuming, requiring manual adjustments and remolding to achieve the correct occlusion. Additionally, wax impressions only sometimes effectively replicate the dynamic aspects of a patient’s bite, leading to inaccuracies in the occlusal assessment. This traditional method relies heavily on the clinician’s skill and experience, which can lead to outcome variability. The traditional method of conducting an occlusal analysis using a facebow involves transferring the spatial orientation of the maxillary arch and occlusal plane to a dental articulator [24,25]. This technique ensures the articulator replicates the patient’s jaw movements and occlusal relationships. The facebow records the relationship between the maxillary arch and a reference point, usually the axis of the temporomandibular joint. The data collected allow for the accurate mounting of casts on the articulator, which is essential for fabricating prostheses or orthodontic appliances that accurately match the patient’s natural occlusion and jaw movements. This method, while accurate, can be time-consuming and relies heavily on clinician skills. In contrast, the new digital method employs intraoral scanning systems, providing greater accuracy and efficiency. It captures precise digital impressions of the mouth, allowing for a more accurate replication of occlusion. This method integrates digital technologies to record mandibular movements and design prosthetic restorations that closely mimic natural dental movements, potentially leading to better functional integration and reducing the need for manual adjustments [24,26]. The digital method offers advantages over traditional techniques, including improved precision, reduced treatment time, and enhanced patient comfort. However, the effectiveness of this method depends on the accuracy of the digital tools and the operator’s expertise. Yue et al. [23] developed a 3D digital smile design technique using virtual articulation for esthetic dentistry. This approach utilized a digital facebow and a virtual articulator to analyze occlusal data and jaw movements, ensuring stable occlusion and smooth jaw patterns. The technique facilitated the design of new prostheses, maintaining stable occlusion and patient satisfaction over 9 months. Sun et al. [24] presented a fully digital workflow for fabricating occlusal stabilization splints. This method used CAD/CAM systems and a digital facebow based on optical sensor technology. The study highlighted the workflow’s clinical feasibility, accuracy, and efficiency compared to traditional methods, demonstrating the potential for improved production and patient care. Chou et al. [25] developed a personalized virtual dental articulator using computed tomography (CT) data and motion tracking. This tool mathematically modeled jaw movements for dental restoration design, replacing traditional facebow transfers. The articulator’s effectiveness was validated by comparing simulation data with actual jaw movement measurements.

Jeong et al. [26] evaluated the accuracy of semi-adjustable articulator contacts compared to intraoral contacts during eccentric mandibular movements. Their study revealed variations in concordance affected by time and whether contacts were on working or nonworking sides. They concluded that while initial eccentric tooth contacts on the articulator were reliable, occlusal adjustments might be necessary post delivery. Prakash et al. [27] conducted a systematic review assessing the utility of the facebow in complete denture fabrication. The review compared facebow use against simplified techniques using anatomical landmarks and found similar clinical efficiency and patient acceptability results. The review called for more research for conclusive results on changing clinical practices. Kubrak et al. [28] compared edentulous patients treated traditionally and using a face-bow and a Quick Master articulator. The study aimed to establish a simple method for occlusal recording and compare the treatment outcomes of using an articulator and traditional methods in fabricating complete dentures. The study involved 60 patients, with clinical examinations and patient surveys conducted post treatment. The findings suggested that using an articulator in denture fabrication resulted in more physiologic and balanced occlusion, shorter adaptation periods, and positive patient feedback.

Linsen et al. [29] highlighted the significance of registration techniques on condyle displacement and electromyographic activity, illustrating the intricate biomechanics involved in stomatognathic health and the precision required in dental prosthetics. Resende et al. [30] emphasized the role of operator experience, scanner type, and scan size in the accuracy of 3D dental scans, shedding light on the importance of technical expertise and equipment in achieving optimal prosthetic outcomes. Li et al. [31] contributed to this understanding by focusing on the design of occlusal wear facets in fixed dental prostheses, indicating the necessity for personalized approaches in dental restoration to mimic natural mandibular movements. Abdulateef et al. [32] discussed the clinical accuracy and reproducibility of virtual interocclusal records, stressing the potential of digital technologies in enhancing the precision of dental measurements and fittings. Cicciù et al. [33] explored the strength parameters in the “Toronto” osseous prosthesis system, providing valuable insights into dental implant’ mechanical properties and durability. In a later study, Cicciù et al. [34] delved into prosthetic and mechanical parameters affecting the facial bone under the load of different dental implant shapes, further emphasizing the need for a nuanced understanding of biomechanical interactions in implant dentistry. Finally, Resende et al. [30] reiterated the influence of operator experience, scanner type, and scan size on 3D scans, reinforcing the multifaceted nature of factors impacting the precision and reliability of digital impressions in prosthetic dentistry. These studies underscore the multidimensional considerations essential in designing, implementing, and evaluating dental prosthetics and implants.

The need to elaborate occlusal surfaces in the CAD phase that are in harmony with the clinical situation is evident due to the need to produce monolithic restorations that allow for minimal intraoral correction. During scan acquisition, accuracy is related to several factors, such as the device’s or software’s technical characteristics, and is dependent on operator experience. An essential issue in CAD manufacturing is the precision fitting of the scan acquired. The PMS system is efficient and valuable if the prosthesis is made to the required vertical dimension, with the upper and lower scans assembled correctly. By this, Jae-Min Seo proposes checking the accuracy of scan fitting using scan acquisition with articulation card markers, a technique integrated into our study [31]. However, compared to the procedure described by Jae-Min Seo, there is no adjustment of the position through a post-elaboration modification. We are conscious of the various problems that can occur during bite detection checks, such as occlusal interpenetration or mandibular distancing, as noted by Abdulateef et al. [32].

Saraa Abdulateef shows frequent compenetration of fitting, with the possibility of under-occluded artefacts. This phenomenon seems related to the compressibility of the periodontal ligament in MI. For this reason, our study decided to begin the observation by detecting clinical contact areas [31] and following artefacts with a slight increase of 25 µ in an area of 0.48 mm in the occlusal contact zones.

The investigation showed that the prosthesis was correct in 77% of cases, with 12.5% requiring subtractive modifications and 10% requiring additive modifications, with a minimum incidence of 3% corrections in excursive areas. This differs from Li’s research, which does not identify the effectiveness of PMS use. In Li’s study [31], the amount of occlusal correction of the tooth surface was assessed by comparing overlapping scans of crowns placed before and after the occlusal adjustment one month later; the authors report both qualitative and quantitative data and conclude that there are no statistically significant differences between PSM fabrication and standard fabrication; however, the use of PSM showed a lower error. There is no indication in Li’s article regarding the necessary control of the fitting of the scans, as we carried out in our audit by comparing the occlusal contacts detected at the time of scanning with the articulation chart and the digitally acquired contacts; this may have influenced the degree of occlusal adjustment required in their work to achieve correct occlusal integration at maximum intercuspation, which is independent of whether or not the PMS was used [31]. The PMS is effective in decreasing potential contacts during the excursion phase. It does not correct possible errors due to the fitting of the scans. For this reason, it is beneficial to check the fitting of the scans by analyzing marks reproduced using the articulation table. 

### Limitations

The main limitation of this study includes the lack of a control group and the relatively small number of patients treated. A sample size calculation was not possible due to the novelty of the approach. This limited the study’s capacity to comprehensively compare the new fully digital protocol with traditional methods and generalize the findings. The results, therefore, are preliminary and suggest a need for further research with larger sample sizes and control groups for a more robust evaluation of the protocol’s efficacy. Extending the protocol to larger-span bridges could be feasible too, but it would require additional research and validation to ensure accuracy and effectiveness. The specific characteristics of larger spans, such as increased complexity and the potential for more significant occlusal forces, would need to be considered in future studies.

## 5. Conclusions

In conclusion, this clinical audit introduces a pioneering digital protocol for recording occlusal analyses in prosthodontic rehabilitation. By integrating intraoral scanning systems with CAD software and leveraging the Patient Specific Motion (PSM) tool, we achieve precise occlusal replication and functional integration, surpassing traditional methods in efficiency and accuracy. This study’s innovative approach minimizes the need for manual occlusal adjustments, demonstrating the potential of digital technologies to enhance prosthetic outcomes significantly. However, this study’s limitations include the absence of a control group, a relatively small patient sample, and the application of the protocol within a specific clinical context, which may restrict the generalization of the findings. The reliance on advanced digital tools also underscores the necessity of operator expertise, emphasizing the importance of comprehensive training in successfully implementing the protocol. Future research should aim to validate these findings through larger, controlled studies, explore the protocol’s applicability across a broader range of dental restorations, and investigate the integration of emerging technologies to refine the occlusal analysis and rehabilitation processes further. This research trajectory promises to elevate the standards of prosthodontic care and expand the boundaries of digital dentistry.

## Figures and Tables

**Table 1:** CAD parameters.

Dental Units	Software	Level of Influence (CAD)	Radius (CAD)	Occlusal Adjustments in MI	Occlusal Adjustment during Lateral Movements	Occlusion	Prototype Models
52	3Shape	1 DA 25 µ	0.48 mm	Accufilm 21 µ	Accufilm 21 µ	MI	No

**Table 2:** Distribution of the restorations.

	Single	Multiple
Teeth	32	3 (3 units)
Implants	8	1 (3 units)
	40	4 (12 dental units)

**Table 3:** Number and type of finishings.

Restorations	No Finishing	Subtractive Finishing	Addictive Finishing	Dinamic Finishing
52	39–75%	9–17.3%	4–7.7%	7–13%
	30 single teeth, supported	2 3-unit teeth, supported	2 single teeth, supported	2 single teeth, supported
	6 single implants, supported	1 3-unit implant, supported	2 single implants, supported	1 3-unit implant, supported
	1 3-unit implant, supported			2 single-implants, supported

## Data Availability

Data are available in the manuscript and are reproductible.
